# Recognition of nonself is necessary to activate *Drosophila*’s immune response against an insect parasite

**DOI:** 10.1186/s12915-024-01886-1

**Published:** 2024-04-22

**Authors:** Alexandre B. Leitão, Ramesh Arunkumar, Jonathan P. Day, Nancy Hanna, Aarathi Devi, Matthew P. Hayes, Francis M. Jiggins

**Affiliations:** 1 Department of Genetics, University of Cambridge, Cambridge, UK; 2 Champalimaud Foundation, Lisbon, Portugal; 3 Royal College of Surgeons in Ireland, Dublin, Ireland; 4 Department of Zoology, University of Cambridge, Cambridge, UK

**Keywords:** Immune recognition, Parasitoid wasps, *Drosophila melanogaster*

## Abstract

**Background:**

Innate immune responses can be activated by pathogen-associated molecular patterns (PAMPs), danger signals released by damaged tissues, or the absence of self-molecules that inhibit immunity. As PAMPs are typically conserved across broad groups of pathogens but absent from the host, it is unclear whether they allow hosts to recognize parasites that are phylogenetically similar to themselves, such as parasitoid wasps infecting insects.

**Results:**

Parasitoids must penetrate the cuticle of *Drosophila* larvae to inject their eggs. In line with previous results, we found that the danger signal of wounding triggers the differentiation of specialized immune cells called lamellocytes. However, using oil droplets to mimic infection by a parasitoid wasp egg, we found that this does not activate the melanization response. This aspect of the immune response also requires exposure to parasite molecules. The unidentified factor enhances the transcriptional response in hemocytes and induces a specific response in the fat body.

**Conclusions:**

We conclude that a combination of danger signals and the recognition of nonself molecules is required to activate *Drosophila*’s immune response against parasitic insects.

**Supplementary Information:**

The online version contains supplementary material available at 10.1186/s12915-024-01886-1.

## Background

Organisms must be able to reliably detect when they are infected to mount an appropriate immune response, and this frequently relies on the recognition of nonself. In adaptive immune systems, receptors generated somatically by gene rearrangement and mutation can recognize virtually any pathogen-derived antigen. In the case of innate immune systems, pattern recognition receptors (PRRs) detect pathogen-associated molecular patterns (PAMPs). These are typically molecules such as flagellin or peptidoglycan that are absent from the host but highly conserved across a broad class of pathogens [1]. An alternative way to sense infection is to detect danger signals such as cell damage. Here, damaged cells release damage-associated molecular patterns (DAMPs), which bind host receptors and trigger the immune response [2]. Finally, pathogens may be detected by the host innate immune system because of “missing self”—they lack some factor found on host cells that inhibits immune activation [3, 4].

Sometimes, immune responses must be mounted against parasites that are related to the host. For example, plants can be infected by other plants, insects by other insects, and some mammals are even infected by transmissible cancer cells derived from their own species [5]. Innate immune receptors can evolve to recognize such parasites. For example, tomato plants have evolved a PRR to recognize a PAMP produced by the pathogenic plant *Cuscuta reflexa* [6, 7], and Toll-like receptors in mammals can recognize snake venom [8] However, it is unclear whether this will be more broadly true. In many cases, it may be difficult to recognize PAMPs, as there will be fewer conserved differences between related pairs of hosts and pathogens. This problem may be exacerbated as there is selection on the pathogen to escape recognition by losing their PAMPs, and this may be easier to evolve if you are already similar to your host. If this is the case, recognition may rely on danger signals or detecting missing self.

This problem is especially acute for insects as they are frequently parasitized by other insects [9]. Many parasitoid wasps infect their insect hosts by injecting eggs into their haemocoel. Typically, infection by parasitoids leads to activation of an immune response that involves the formation of a cellular capsule around the parasitoid egg that later becomes melanized [10–12]. The cellular immune response to parasitoid wasps in *Drosophila melanogaster* involves the differentiation of a hemocyte type rarely found in healthy larvae, the lamellocyte [10]. These form the outer layer of the cellular capsule around the parasitoid egg, which is melanized when pro-phenoloxidase 2 and 3 (PPO2 and PPO3) are activated in crystal cells and lamellocytes, respectively [13].

The recognition of parasitoid infections relies in part on danger signals. Lamellocyte differentiation can be triggered by sterile wounding of the larval cuticle [14], which presumably mimics a parasitoid piercing the cuticle with its ovipositor. Furthermore, in many insects, introducing inert objects into the haemocoel leads to a cellular encapsulation response [15, 16], and in species such as *D. yakuba*, this is accompanied by the object being melanized [17, 18]. However, in other cases, these danger signals only lead to an incomplete immune response, as a cellular capsule forms, but there is only a low level of melanization [18, 19]. Interestingly, *D. melanogaster* larvae that have been parasitized by the wasp *Asobara tabida* are more likely to strongly melanize inert objects [19]. This suggests that a molecule injected by the parasitoid might activate the melanization response. Studies of flies that mount an autoimmune response in which their own tissues are melanized suggest that missing self may be an important factor in guiding this immune response. The flies’ own tissues are melanized if they are not protected by extracellular matrix [20], and this relies on the glycosylation of proteins in the matrix [21]. Therefore, if parasitoids lack these modifications to their surface proteins, this may provide a mechanism by which they are recognized by the immune system.

The transcriptomic responses following infection provide insights into how immune responses and other pathways are activated. This encompasses reactions to both wounding and the recognition of the pathogen. For example, when *D. melanogaster* larvae were inoculated with bacteria, changes in gene expression are induced both by the recognition of bacterial molecules and wounding alone [22]. These responses can also be manipulated by parasite molecules. For example, the transcriptional response of *Drosophila* to two closely related parasitoid wasp species differs greatly, likely because of different compositions of the venoms they produce [23]. Tattikota and colleagues compared the effects of *Leptopilina boulardi*-infection and injury using single-cell RNA sequencing of hemocytes and found that wounding and infection both result in the production of mature lamellocytes and cells producing antimicrobial peptides [24]. However, as *L. boulardi* wasps inject eggs and venom simultaneously during infection, it is not possible to know which of these elicits the transcriptional response in these experiments. Furthermore, the melanization of the wasp eggs relies on a humoral immune response involving the secretion of molecules from the fat body [25] and it is unknown how this is regulated. Therefore, the triggers of the transcriptomic response to parasitoids remain unknown.

Here, we examine how the combination of danger signals and nonself activates different components of the immune response of *D. melanogaster* to parasitoid wasps. Combining physiological measurements with transcriptional profiling of two immune tissues (hemocytes and fat body), we describe how both danger signals and nonself can both trigger the cellular immune response, but nonself is required for the humoral response and melanization. This suggests the presence of a currently unknown mechanisms of immune recognition that play a critical role in defense against this important group of insect parasites.

## Results

### Danger signals induce immune cell differentiation

**Fig. 1:**
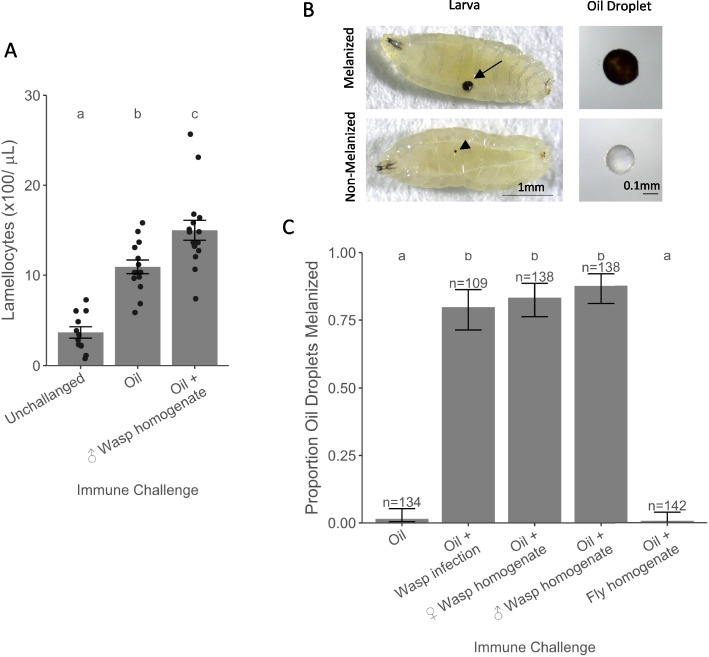
The effect of parasitoid wasp exposure on lamellocyte differentiation and the melanization of oil droplets. **A** Concentration of lamellocytes in the hemolymph of unchallenged larvae and larvae 48 h postinjection with oil or oil + wasp homogenate. The data points are independent measurements of hemolymph pooled from 8 to 10 larvae. **B** Oil droplets injected into larvae are either melanized (arrow) or not. Melanization of the cuticle resulting from injection wounding is often visible (arrowhead). **C** Proportion of larvae with melanized oil droplets 48 h after different immune challenges. Different letters represent treatments with statistically significant differences (Tukey’s honest significant difference test, *p* < 0.01)

In *D. melanogaster*, parasitoid wasp attack induces the rapid differentiation of blood cells called lamellocytes, which encapsulate and melanize the wasp. To discern the signals required to induce lamellocyte differentiation and melanization, we performed a series of injections into larvae of a wild-type and outbred population. It has previously been reported that sterile wounding of the larval cuticle induces lamellocyte differentiation [14]. In line with this, we found that injecting a droplet of paraffin oil induced lamellocyte differentiation (Fig. 1A; main effect of treatment: *F* = 36.187, d.f. = 2, 38, *p* = 1.59 × 10^−9^; oil vs. control *t* = 5.298, d.f. = 38, *p* < 1.57 × 10^−5^). We have previously shown using the same host and parasite genotypes that wasp infection results in that wasp infection results in only slightly more lamellocytes at this timepoint, suggesting this is a physiologically relevant response (~ 22 × 100 lamellocytes/µl) [26].

To examine the role of parasite molecules in this response, we homogenized adult male wasps in paraffin oil before injecting the fly larvae. Therefore, this treatment combines both wounding from injection and exposure to parasite molecules. We found that the addition of wasp homogenate led to a larger number of lamellocytes being produced (Fig. 1A; oil vs. wasp homogenate *t* = 3.26, d.f. = 38, *p* = 0.007). This suggests that danger signals resulting from the wound created during injection is the primary factor triggering lamellocyte differentiation, but the response is amplified by recognition of nonself wasps. Similar triggers may be important in nature, as wasps must pierce the larval cuticle with their ovipositor when parasitizing a larva.

### Parasite molecules activate the melanization response

The final step of the immune response against parasitoid wasps is the melanization of the wasp egg. To test if this requires the recognition of nonself, we examined whether paraffin oil droplets were melanized 48 h after injection (Fig. 1B). Wounding alone was not sufficient to activate this response, as paraffin oil by itself did not induce a strong melanization reaction (Fig. 1C). However, if larvae were previously infected by a low virulence *Leptopilina boulardi* strain (G486), the melanization of the oil droplet increased (Fig. 1C; main effect of treatment: *Χ*^*2*^ = 577.39, d.f. = 4, *p* < 2 × 10^−16^; oil vs. oil + wasp infection: *z* = 7.612, *p* = 2 × 69^−13^). Therefore, the presence of the parasite is required to trigger this immune response.

To test whether parasitoid wasp molecules are responsible for the activation of the immune response, we injected flies with paraffin oil containing homogenized female wasps. This led to a robust melanization response that was indistinguishable from that seen when the flies had been parasitized (Fig. 1C; female wasp homogenate vs oil: *z* = 7.967, *p* = 1 × 55^−14^). Furthermore, this reaction is not due to the presence of eggs or venoms in the female wasp homogenates, as male wasp homogenates induced a similar response (Fig. 1C; male homogenate vs female homogenate: *z* = 1.051, *p* = 1).

The immune response to our crude homogenate of parasitoid wasps could be a specific response to molecules in the parasitoid tissue or a general response to injecting damaged cells, which are known to release DAMPs that activate the innate immune system [27]. To test these hypotheses, we injected larvae with paraffin oil containing *D. melanogaster* homogenate. This did not induce melanization (Fig. 1C; oil vs fly homogenate: z =  − 0.634, *p* = 1). Together, these results indicate that the *D. melanogaster* immune system recognizes nonself molecules in the parasitoid wasp to activate the melanization response.

To determine if the fly immune system recognizes wasp proteins, we treated the crude wasp homogenate with pronase, a mixture of different proteases extracted from the extracellular fluid of the bacteria *Streptomyces griseus*. Pronase treatment reduces the ability of the wasp extract to induce melanization, when the treated extracts are tested at lower concentrations (Additional file 1: Figure S1, main effect of pronase treatment: *Χ*^*2*^ = 47.49, d.f = 1, *p* = 5.54 × 10^−12^). However, when this experiment was repeated with proteinase K, a broad range serine protease [28], this did not reduce the proportion of melanized oil droplets (Additional file 2: Figure S2, main effect of proteinase K treatment: *Χ*^*2*^ = 0.002, d.f = 1, *p* = 0.96). Notably, this result held across a dilution series of the wasp extract, suggesting this is not simply because the proteinase K digestion is incomplete, leaving enough concentration to trigger the melanization response. Serendipitously, we tested samples where wasps were autoclaved before homogenization. In itself, this has no effect on melanization rates (autoclave vs. no autoclave: *z* = 1.254, *p* = 1). However, when the autoclaved homogenate was treated with proteinase K, the number of melanized oil droplets was significantly reduced (autoclave vs. autoclave + proteinase K: *z* = 5.41, *p* = 6.3 × 10^−7^). Together, these results suggests that one or more proteins in the wasp body contribute to the activation of the fly immune system.

### Exposure to parasitoid molecules activates the humoral immune response

In addition to the cellular immune response, the melanization of the capsule formed around wasp eggs relies on a humoral immune response involving the secretion of molecules from the fat body [25]. To understand the effects of danger signals and wasp molecules on this response, we sequenced RNA extracted from the fat body 24 h postinjection of paraffin oil, paraffin oil with wasp homogenate, and noninjured controls (unchallenged). This timepoint was selected as it is when the capsule is forming and many humoral immune genes are upregulated [29]. After aligning the RNA-seq reads to the *D. melanogaster* genome, the number of uniquely mapped exonic reads ranged from 3,288,236 to 15,962,793 (Additional file 3: Table S1).

**Fig. 2:**
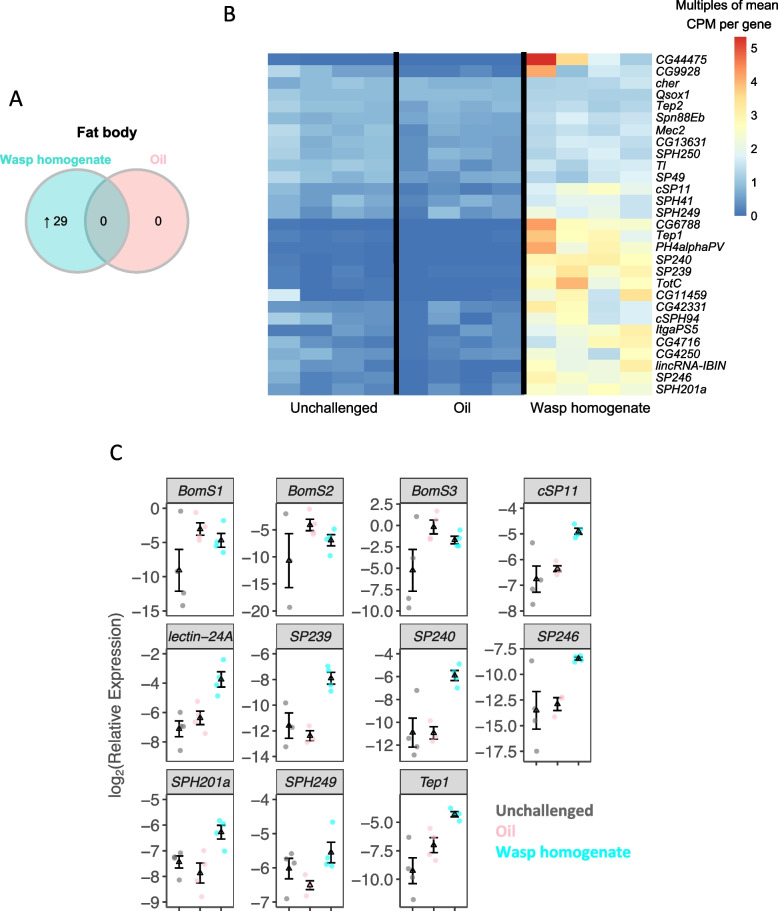
Transcriptional response of humoral immune genes to wasp exposure larvae were injected with oil, wasp + oil, or unchallenged. RNA from the fat body was sequenced 24 h post treatment. **A** The number of genes with significant changes in expression compared with unchallenged conditions. **B** The expression of 29 genes with significant changes in expression after immune challenge. **C** Expression of selected genes in the fat body measured by quantitative PCR 24 h after injection with oil droplets or wasp + oil droplets. For each treatment, 4 pools of 5–7 larvae were used to extract RNA

The injection of paraffin oil alone did not lead to the significant upregulation of any genes at 24 h, but the addition of wasp homogenate upregulated 29 genes (Fig. 2A, B). Ten of these encode serine proteases with a trypsin domain (Additional file 4: Table S2), a class of proteins known to be involved in the melanization cascade and Toll pathway [25]. Other upregulated genes encode immunity-related molecules, including Toll, thioester-containing proteins (TEPs), and fibrinogen.

To confirm the specific response to wasp homogenate, we chose a subset of the known or likely immune genes and analyzed gene expression with qPCR under the same experimental conditions as the RNAseq experiment. Inspection of the RNAseq data revealed that some genes, such as some members of the *Bomanin* family, were upregulated by injury alone but fell below the threshold of statistical significance. Representatives of these were included to test if there is a humoral response to injury. This revealed that wounding and the wasp molecules elicit distinct humoral immune responses at 24 h post-infection. The injection of paraffin oil alone was sufficient to upregulate Bomanin genes, which encode short peptides that play a role in killing bacterial pathogens [30] (Fig. 2C; unchallenged vs oil: *p* = 0.04, *p* = 0.05, and *p* = 0.10). However, other genes, including secreted serine proteases, lectin, and Tep, were specifically upregulated in the presence of the wasp homogenate (Fig. 2C). This suggests that a humoral immune response against bacteria can be triggered by wounding, but PAMPs are required for the specific anti-parasitoid response. This may reflect different pathways being activated by injury and wasp homogenate, as Bomanin genes are regulated by the Toll pathway [31], while Tep genes are under the control of both the Toll and JAK-STAT pathways [32].

### Parasitoid wasp molecules amplify the transcriptional response of immune cells to danger signals

**Fig. 3:**
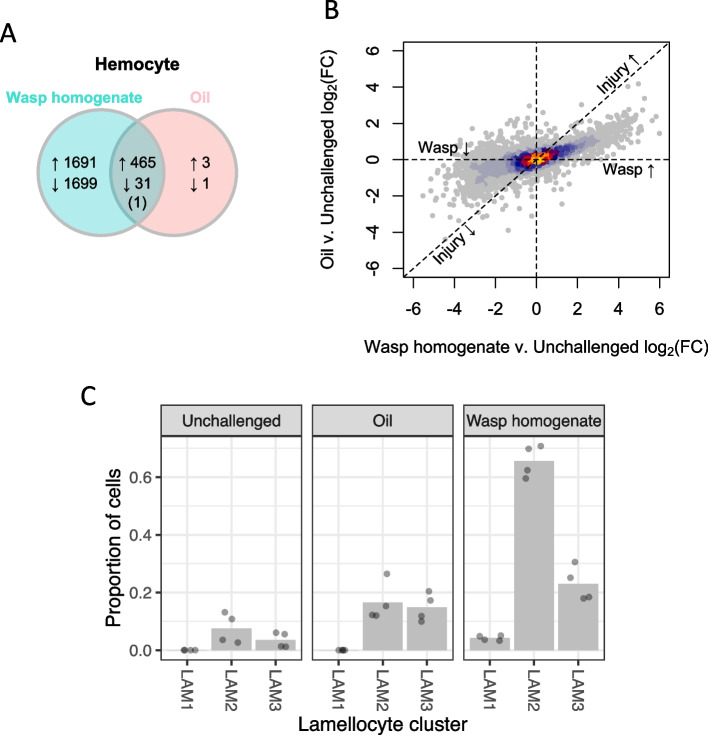
Transcriptional response of hemocytes to wasp exposure larvae were injected with oil, wasp + oil, or unchallenged. RNA from the hemocytes was sequenced 24 h post treatment. **A** The number of genes with significant changes in expression compared with unchallenged conditions. **B** Changes in gene expression induced by injection of wasp homogenate (*x*-axis) and by injection of oil (*y*-axis). Because both treatments cause injury, genes solely regulated by injury will be close to the dashed diagonal 1:1 line. Genes specifically activated by wasp PAMPs will be on the *x*-axis. Relative expression is represented as log_2_ (fold change). **C** Inferred proportion of immature (LAM1 and LAM2) and mature (LAM3) lamellocytes estimated from the RNA-seq data using digital cytometry. Each point is an independent sample, and the bars are the mean

To understand the role of wasp molecules in the cellular immune response, we repeated the RNA-seq experiment on hemocytes. The number of uniquely mapped exonic reads ranged from 2,520,358 to 22,036,188 (Additional file 3: Table S1). There was a much broader transcriptional change in hemocytes than in the fat body (Fig. 3A), with 3887 genes being differentially expressed 24 h after larvae were injected with wasp homogenate (Fig. 3A, Additional file 5: Figure S3). The genes that were significantly differentially expressed when larvae were injected with mineral oil alone were largely a subset of these genes (Fig. 3A). The wasp homogenate and mineral oil injections largely caused the same genes to change in expression, but the magnitude of the transcriptional response was greater in the presence of the wasp molecules (Fig. 3B). Therefore, recognition of the parasite amplifies the response to a danger signal.

The massive transcriptional response of hemocytes may reflect the differentiation of lamellocytes, which are rare in homeostasis but increase after wasp infection. Genes upregulated by wasp homogenate were enriched for biological adhesion and cytoskeleton organization, which may be related to the role of lamellocytes in capsule formation and the changes in cell morphology that occur as these cells differentiate (Additional file 6: Figure S4A). We detected a moderate upregulation of genes involved in lamellocyte differentiation following wounding, such as those associated with actin remodeling and biological adhesion (Additional file 6: Figure S4A), consistent with previous studies [22, 24]. Nevertheless, the recognition of wasp molecules substantially amplified this response (Additional file 6: Figure S4A). The upregulated genes were also enriched for endocytosis, macroautophagy, and other immune functions (Additional file 7: Table S3) [33]. In contrast, genes downregulated by wasp homogenate were enriched for extracellular structure organization, a housekeeping function of plasmatocytes (Additional file 6: Figure S4A).

To test whether these transcriptional changes were linked to the differentiation of lamellocytes, we compared our data to previous results we have generated using single-cell RNA sequencing (scRNA-seq), where we defined cell states that we inferred to be either mature or immature lamellocytes (LAM3 versus LAM1 or LAM2) [26]. We found that genes that were highly expressed in lamellocytes were upregulated by injecting wasp homogenate and vice versa for downregulated genes (Additional file 6: Figure S4B). To investigate this further, we estimated the abundance of different hemocyte types using digital cytometry [34]. This is a statistical technique that estimates cell proportions in “bulk” RNA-seq data using the single-cell expression profile [26] as a reference. We estimated that there was a moderate increase in the proportion of lamellocytes following the injection of an oil droplet (Fig. 3C). However, the injection of wasp homogenate led to the differentiation of mature lamellocytes (LAM3; Fig. 3C) together with large numbers of immature lamellocytes (LAM1 and LAM2; Fig. 3C).

### *Drosophila melanogaster* has evolved to recognize parasitoid-specific molecules

**Fig. 4:**
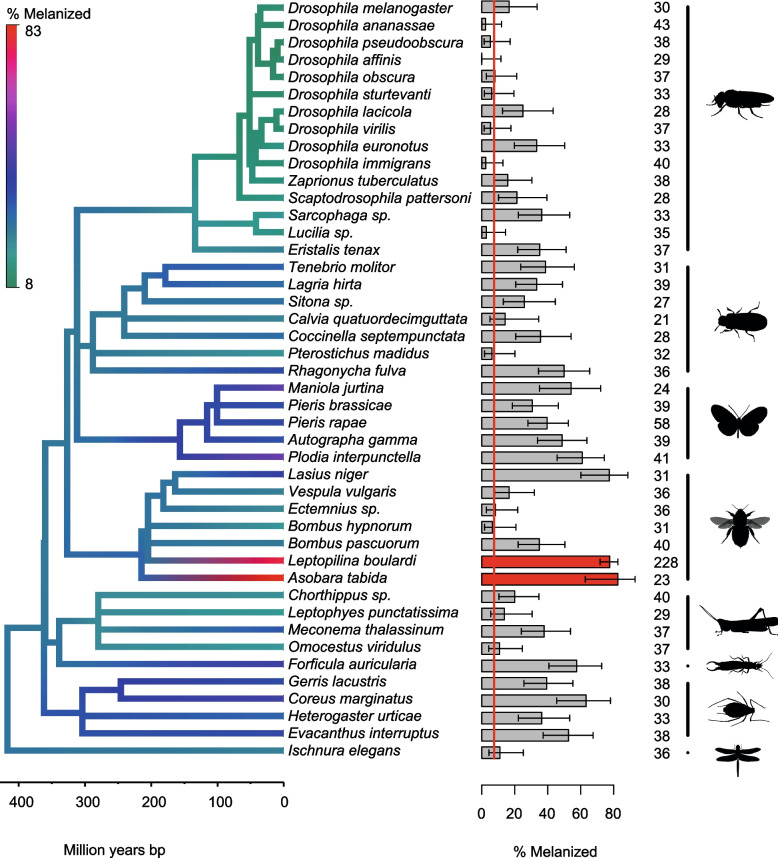
The effect of injecting homogenates of different insects on the melanization of oil droplets. The bar chart shows the proportion of oil droplets that were melanized with 95% binomial confidence intervals. The red line is the rate at which oil droplets were melanized without any insect homogenate (*N* = 241). The red bars are parasitoids of *D. melanogaster*. The tree [35] is colored according to estimated melanization rates from a phylogenetic mixed model in which the parasitoid status was included as a fixed effect. Sample sizes are given beside the bars. The silhouettes represent different insect orders (from top to bottom: Diptera, Coleoptera, Lepidoptera, Hymenoptera, Orthoptera, Dermaptera, Hemiptera, Odonata)

To investigate whether *Drosophila* larvae have evolved to recognize parasitoid-specific molecules, we injected fly larvae with homogenates prepared from 44 insect species and examined whether they activated the melanization response to oil droplets. It is striking that species closely related to *D. melanogaster* do not activate the melanization response (Fig. 4; 95% confidence intervals overlap the red line basal response). However, homogenates of many other insect species cause some level of immune activation (Fig. 4). Therefore, it appears that many insect species contain factors that cause some activation of the melanization response, but *Drosophila* immunity is not activated by self.

The two parasitoid species resulted in the two highest melanization rates of all 44 species. We therefore asked whether this observation could be explained by the rate at which the response changes across the insect phylogeny or whether there was a significant increase in immunogenicity along the parasitoid branch of the tree. We found that the parasitoid-specific response was significantly increased after correcting for the phylogenetic relatedness of the 44 species (Fig. 4; phylogenetic mixed model: *p* = 0.006). Therefore, the *Drosophila* immune system appears to have evolved to respond more strongly to parasitoid molecules.

## Discussion

**Fig. 5:**
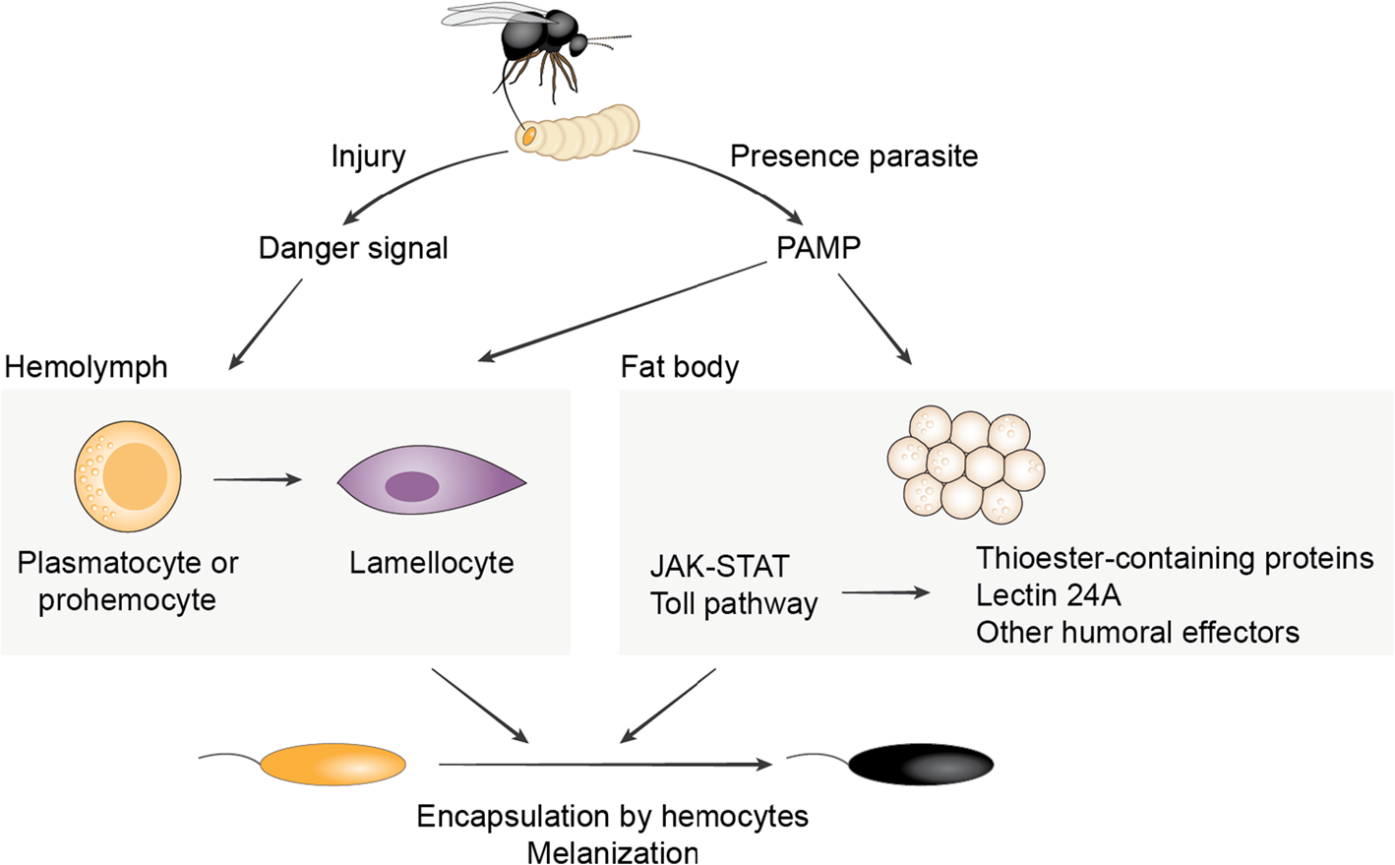
Model of immune activation by parasitoid wasps

Immune responses are multistep processes that require several levels of regulation. Here, we describe how the immune response of *D. melanogaster* against parasitoid wasps is regulated by two modes of immune activation. In line with previous results [14], immune challenge with an inert object induces the differentiation of lamellocytes (Fig. 5). It is also sufficient to result in local melanization at the wound site. The signal required for this is likely a DAMP produced during wounding [36]. However, the final step of the response—melanization of the oil droplet—occurs only when wasp molecules are present (Fig. 5). This suggests that this part of the encapsulation response is activated by recognition of pathogen molecules. This has different effects on the two main immune tissues of *Drosophila*. In hemocytes, it enhances the differentiation of lamellocytes, amplifying the effects of wounding. In the fat body, it triggers the upregulation of a small number of humoral immune genes whose expression is not affected by wounding alone.

Cellular immune responses frequently rely on opsonins that bind to the surface of pathogens and act as a label to direct the cellular immune response. However, this does not appear to explain our results, as we found that sterile oil droplets were melanized if the fly larva had previously been attacked by a parasitoid. Assuming that the wasp molecules were not specifically attached to the surface of the oil droplet, this suggests that they are triggering a systemic response that promotes melanization. This is consistent with our observation that the wasp homogenate upregulates anti-parasitoid factors in the fat body.

Work on *Drosophila* lines that exhibit autoimmunity and melanize their own tissues provides a possible mechanism for how parasitoids are recognized. Epithelial cells are separated from the hemocoel by a thin layer of extracellular matrix called the basement membrane. This protects the fly’s own tissues from being melanized (although when the basement membrane is disrupted, melanization is only triggered if there are other signals such as a loss of cell integrity). This self-tolerance relies on the fly glycosylating extracellular matrix proteins [21]. Therefore, if proteins on the surface of parasites lack these modifications, they may be recognized by the immune system [21]. Therefore, parasitoid recognition may rely on the immune system detecting missing self. Alternatively, there may be direct recognition of wasp molecules. Evidence for a similar effect has come from studies of *Drosophila* larvae exposed to the odor of parasitoids in the environment. The odor is detected by odorant receptors, leading to hemocytes being primed to differentiate into lamellocytes upon infection [37].

If the immune response had evolved to recognize a specific wasp PAMP, one might expect a strong immune response to the parasitoids or their relatives but not to other insect orders. If the response was to missing self, then any insect distantly related to *Drosophila* might trigger a response. The result fell in between these extreme scenarios. The two parasitoids triggered a stronger immune response than any other insect we sampled, suggesting that the immune response can recognize the presence of specific parasite factors. However, insects from other orders were also melanized at high rates. Ultimately, functional studies of the underlying mechanism are needed. If a receptor recognizes a self-associated molecular pattern (SAMP) and activates an inhibitory pathway, the immune response is triggered by the missing self. If a receptor binds wasp proteins and this activates immunity, it may be better classified as nonself-recognition of a PAMP.

We found that homogenates prepared with other *Drosophila* species consistently induce a very poor response. This contrasts with a previous report that shows encapsulation and melanization of fat bodies from heterospecific tissue transplants when the donor is a *Drosophila* species outside the melanogaster group [38]. However, in this case, host larvae are mutants in both *hop*, which causes the differentiation of lamellocytes in homeostasis, and *GCS1*, which glycosylates proteins in the extracellular matrix [21]. Both of these mutations may activate the immune system to respond more strongly to weakly immunogenic objects, possibly explaining the discrepancy with the results presented here.

Combining nonself recognition with danger signals can allow immune systems to mount an appropriate response, for example, preventing immune responses to harmless self, such as a mammalian fetus, or enhancing responses to dangerous self, such as cancer [39]. The reason why the anti-parasitoid immune response relies on both danger signals and nonself recognition is a matter for speculation. However, the recognition of wasp molecules may be used to adjust the immune response to target this specific parasite, as wounding may occur for many other reasons. This regulation step may be important for the fly to minimize costs associated with the activation of the melanization cascade. Artificial activation of the melanization response is detrimental for host tissue physiology [40], while the production of lamellocytes, which is enhanced by wasp molecules, is thought to be energetically costly for the fly [41]. Without this step of immune regulation, any cuticular wound might result in activation of the melanization cascade and incur associated costs.

All major macromolecules, proteins, carbohydrates, nucleic acids, and lipids can act as PAMPs (1), and the crude wasp homogenate used in our experiments includes all these molecules. However, we found that treatment with proteinases reduces the activity of the PAMP, suggesting that the active molecule may be a protein or glycoprotein. While pronase efficiently abolished the immunogenic activity of the wasp homogenate, proteinase K was only effective if the wasps were autoclaved prior to homogenization. This could indicate that the wasp protein may be resistant to proteinase K digestion [42, 43], and high temperature treatment may have modified the configuration of the wasp PAMP and made it accessible to proteinase K. Alternatively, there may be redundancy among different immune elicitors in the wasp homogenate, with microbes or carbohydrates being abolished by autoclaving and proteins being eliminated by proteinase K. The nature of the immunogenic protein is unknown. For example, it could act as a classical PAMP recognized by a pattern recognition receptor, or it could be a molecule such as a serine protease that triggers a response due to its biological activity.

PAMPs play a central role in activating the insect immune response against microbes. In some cases, the downstream responses may be similar to the anti-parasitoid response—for example, the bacterial cell wall component peptidoglycan can trigger the melanization in silkworms [44]. An unanswered question is the extent to which the pattern recognition molecules and signaling pathways are conserved across these different responses.

## Conclusions

In conclusion, we show that *Drosophila*’s immune system can recognize the presence of wasp parasites and uses this recognition to modulate the cellular and humoral responses that are initiated by injury. Deciphering the molecular basis of this recognition promises to reveal novel components of the insect innate immune system.

## Methods

### *D. melanogaster* and *Leptopilina boulardi* maintenance

For all experiments except RNAi, we used an outbred *D. melanogaster* population that was established from 372 isofemales caught in Cambridgeshire, UK, in October 2017. Population size was maintained over 500 flies per generation and had over 10 generations of laboratory adaptation before the start of experiments. For experimental procedures, flies were allowed to lay eggs overnight on agar plates covered with yeast (*Saccharomyces cerevisiae*—Sigma YSC2). Eggs were washed from the agar plate with PBS and transferred into 1.5 ml microcentrifuge tubes. Then, 13 μl of eggs and PBS (~ 150 eggs) were transferred onto 50 mm cornmeal food plates. These were incubated for 72 h before experiments. Developing and adult *D. melanogaster* were maintained at 25 °C and 70% relative humidity in an 8 h–16 h dark–light cycle.

*Leptopilina boulardi* was maintained by allowing females to infect 1st instar larvae of the outbred population and incubating them at 25 °C. Adult wasps were collected 24 days after infection and maintained at room temperature with a drop of honey for a maximum of 5 days before infection. To infect *D. melanogaster* larvae, 3 *L. boulardi* females were allowed to infect larvae on cornmeal food plates for 3 h.

### Insect species

We used 44 species of insects to test whether they activated the melanization response. Drosophilid species (kind gift from Ben Longdon), *Asobara tabida* strain SFA3 (collected in Sainte Foy-Lès-Lyon, Rhône, France in 2012 and provided by Fabrice Vavre), and *L. boulardi* strain G486 [45] were laboratory-maintained stocks. All other species were collected in Cambridge, UK, in July 2018 and identified morphologically. For large species, a single specimen was collected, while for smaller species, multiple individuals were pooled.

### Oil injections

To test whether insect extracts could activate the immune response, we homogenized insects in paraffin oil. Our initial characterization of wasp extracts used 20 female *L. boulardi* in 200 μl of paraffin oil (Sigma Aldrich M5904; approximately 0.025 mg wasp/μl oil). In the experiment involving multiple species, specimens were weighed, and paraffin oil was added to reach a concentration of 0.025 mg/μl. For large specimens, the thorax was used, while for small specimens, the entire animal was used (body part did not have a significant effect on melanization rates). Specimens were homogenized in paraffin oil with a pestle in 0.5 ml microcentrifuge tubes. To remove large particles, the solution was centrifuged for 1 min at 300 g, and the supernatant was transferred to a new 0.5 ml microcentrifuge tube.

Borosilicate glass 3.5″ capillaries (Drummond Scientific Co. 3–000-203-G/X) were pulled to form thin needles in a needle puller (Narishige PC-10). The needle was backfilled with the oil solution with a syringe and attached to a nanoinjector (Drummond Scientific Co. Nonoject II). Late 2nd instar and early 3rd instar larvae were carefully removed with forceps from cornmeal food plates and placed on filter paper in groups of 10. Larvae were carefully injected with 4.6 nl of solution. After injection, ddH_2_O was added with a brush to remove the larvae, and 40 larvae were transferred into a cornmeal food vial at 25 °C, 70% relative humidity, and an 8:16 dark to light cycle. After 48 h, larvae were removed with a 15% w/v sugar solution and scored for total melanization of the oil droplet.

### Hemocyte counts

To count hemocytes, larvae were injected as described above. After 48 h, injected and control larvae were collected, washed in PBS, dried on filter paper, and pooled in groups of 8 to 10 larvae in a well of a multiwell porcelain plate. Larvae were rapidly dissected with a pair of forceps from the ventral side. Hemolymph was recovered with a 1–10 μl micropipette and transferred into a 0.5-ml microcentrifuge tube. One microliter of hemolymph was collected, diluted in 9 μl of neutral red solution (1.65 g/L PBS—Sigma–Aldrich N2889) and thoroughly mixed. The hemolymph dilution was transferred into a counting Thoma chamber (Marienfeld #0640711), and hemocytes were counted in a total volume of 0.1 μl with a × 40 objective (Leica DM750). Lamellocytes were distinguished from plasmatocytes and crystal cells by morphology.

### Protease treatments

For proteinase K digestion (Merk #70,663), samples were incubated in Tris–HCl 10 mM, pH 8 + CaCl_2_ 3 mM. For pronase (Merk #1,074,330,001), digestion samples were incubated in Tris–HCl 100 mM pH 8, + 10 mMCaCl_2_. In both cases, samples were incubated for 4 h at 50 °C, followed for 15 min at 94 °C to inactivate proteases.

### RNA sequencing

We performed RNA-seq on flies injected with wasp homogenate or oil droplets and unchallenged flies. Hemocytes from ~ 100 larvae were pooled in 100 μl of PBS 24 h after injection. Fat body samples were dissected from 8 third instar larvae and pooled in 100 μl of PBS. RNA was purified from hemolymph or fat body samples in an identical manner: 1 ml TRIzol [Ambion: 15596018] was added to collected tissue, and the samples were homogenized by pipetting several times. Two hundred microliters of chloroform [Fisher Scientific: C/4920/08] was added; samples were shaken for 15 s, incubated at room temperature for 3 min and then centrifuged at 12,000 g for 10 min at 4 °C. The upper aqueous phase (approximately 500 μl) was removed to a fresh tube, and RNA was precipitated by adding 2.5 volumes of isopropanol and incubated at – 20 °C for 1 h. RNA was pelleted by centrifugation, washed with 70% ethanol and resuspended in 15 μl of nuclease-free water [Ambion: AM9930]. RNA was quantified by Qubit fluorometer 2.0 [Thermo Fisher Scientific: Q32866] with the Qubit RNA HS Assay Kit [Thermo Fisher Scientific: Q32852], and integrity was assessed by gel electrophoresis. A total of 100–4000 ng of RNA was used for RNA-Seq library preparation.

Libraries were prepared using the KAPA Stranded mRNA-Seq Kit Illumina® platform. The TrueSeq DNA Low Throughput adaptors used were from the Illumina® TruSeq™ KAPA Si adaptor kit KK8701, and the adaptor concentrations and the number of PCR cycles used to amplify the final libraries were adjusted to the total amount of RNA used for each library. Seven hemocyte libraries that gave a low final concentration (< 2 ng/μl) were reamplified for four more cycles. Quality control of the libraries to ensure that no adapter dimers were present was carried out by examining 1 μl of a 1:5 dilution on a High Sensitivity DNA chip (Agilent Technologies: 5067–4626) on an Agilent 2100 Bioanalyzer. The average library size including adapters was 350 bp. Sequencing was carried out at the Cancer Research UK Cambridge Institute in June 2019. All 24 libraries were multiplexed and sequenced on one lane of HiSeq4000 using 50 bp single end reads.

### Differential expression tests

Sequenced RNA-seq reads were trimmed and aligned to the *D. melanogaster* genome, and read counts per gene were estimated. Using Trimmomatic v.0.36 [46], we clipped adaptor sequences, removed the first three and last three bases, filtered strings of low-quality bases found in 4 bp sliding windows where quality dropped below 20, and ensured that the remaining reads had a minimum size of 36 bp. We mapped the resulting reads using STAR v2.6 [47] to the *D. melanogaster* reference (r6.28) [48] attained from Flybase (FB2019_02) [49]. We prepared the genome for STAR mapping using a sjdbOverhang of 49. Then, we mapped reads using the basic option for the twopassMode parameter, filtered multimapped reads, and sorted the remainder by coordinates. We used featureCounts [50] to compute read counts for genes using their FlyBase IDs. We only considered reads with a minimum mapping quality score of 10.

We performed differential expression tests for the fat body and hemocyte libraries separately using edgeR v.3.24.3 [51, 52] and limma v.3.38.3 [53]. We only kept genes that had CPM greater than or equal to 2 in at least four samples for a given tissue. We normalized read counts using the trimmed mean of *M*-values. For a given tissue, we had four replicate libraries for each of three groups: wasp homogenate, oil, and unchallenged. Salivary gland and male germ tissues were difficult to exclude completely when dissecting larvae and isolating the fat body of *D. melanogaster*. To minimize noise in our differential expression tests attributable to this limitation, we excluded genes that had enriched expression in the aforementioned tissues. We obtained tissue-level RNA-seq expression data from FlyAtlas2 [54] and calculated the tissue specificity index (Tau) [55] for each gene in the larvae and adult males separately. We then excluded tissue-specific fat body expressed genes (Tau > 0.8) with the greatest expression either in the larval salivary gland or adult male testes. We only excluded genes that had FPKM > 1 in either of those two tissues in FlyAltas2. We fit a linear model using limma contrasting gene expression among the three groups (full model in script). After checking that the mean–variance trends followed the expected dispersion, we fit contrasts and used the *eBayes* function to uncover genes with evidence of significant differential expression between the wasp homogenate and unchallenged comparison and the oil and unchallenged comparison separately. We used the *heatscatter* function from the LSD v.4 R package to compare the log_2_FC in expression between the two comparisons. We then extracted genes with *P* values of less than 0.05 after a false discovery rate (FDR) correction. For a few of these genes, we divided counts per million reads (CPM) for each library by the overall total across all libraries per gene to compare expression levels across samples. We plotted a heatmap of relative gene expression using pheatmap v.1.0.12 R package. Serine proteases were named according to [56]. Log_2_CPM counts for all genes (unfiltered) in hemocyte and fat body tissues are accessible from the following gene expression browser https://arunkuma.shinyapps.io/waspapp/ (last accessed June 2022). We performed gene ontology (Huang et al., 2009) enrichment analyses on the differentially expressed genes using Flymine [57]. The genes detected in each tissue were used as the background list. Nonredundant gene ontology terms were identified using REVIGO [58], keeping FDR *P* values < 0.05 and similarity = 0.4.

### Bulk RNA-seq deconvolution

We used the digital cytometric method CIBERSORTx [34] to infer the proportion of hemocyte clusters, which were identified in [26], in the bulk RNA-seq data. We first created a signature matrix using read counts from 2000 highly variable genes in the scRNA-seq. Between 300 and 500 genes were used for barcoding cell types, and a *q*-value of 0.01 was used to test for the significance of differential gene expression. Quantile normalization was disabled as recommended, and a maximum conditional number of 999 was used by default. Only genes with an average log_2_ expression of 0.5 were analyzed. Five replicates were used to build the scRNA-seq reference file. Half of the available gene expression profiles were randomly selected to generate the file. Then, we imputed cell fractions using the bulk RNA-seq read counts from hemocyte libraries with an S-mode batch correction and used 100 permutations to assess the significance of cluster inferences.

### Gene expression by qPCR

To analyze gene expression by qPCR, RNA was extracted from the fat bodies of 5–7 larvae 24 h postinjection. Fat bodies were homogenized in 250 μl TRIzol [Ambion 15596018] with ~ 10 1.0-mm zirconia beads [Thistle Scientific] in a tissuelyser [Retsch MM300] and kept at – 80 °C. For RNA extraction, samples were defrosted and centrifuged for 10 min at 4 °C at 12,000 g. A total of 160 μl of supernatant was transferred into 1.5-ml microcentrifuge tubes, 62.5 μl of chloroform [Fisher Scientific C/4920/08] was added, and the tubes were shaken for 15 s and incubated for 3 min. After a 10-min centrifugation at 12,000 g at 4 °C, 66 μl of the aqueous phase was transferred into a 1.5-μl microcentrifuge tube, 156 μl of isopropanol [Honeywell 33539] was added, and the solution was thoroughly mixed. After 10 min of incubation, the samples were centrifuged for 10 min at 12,000 g at 4 °C, and the supernatant was removed. RNA was washed with 250 μl 70% ethanol and centrifuged for 2 min at 12,000 g at 4 °C. Ethanol was removed, the samples were dried, 20 μl of nuclease-free water [Ambion AM9930] was added, and the samples were incubated at 45 °C for 10 min. cDNA was prepared from RNA samples with GoScript reverse transcriptase (Promega) according to the manufacturer’s instructions. cDNA was diluted 1:10. Exonic primers for *D. melanogaster* immunity genes were designed in the NCBI Primer-BLAST online tool (Additional file 8: Table S4). The gene *RpL32* was used to normalize gene expression (RpL32_qPCR_F-d: 5′-TGCTAAGCTGTCGCACAAATGG-3′; RpL_qPCR_R-h 5′- TGCGCTTGTTCGATCCGTAAC-3′; [59]). The Sensifast Hi-Rox SyBr kit [Bioline, BIO-92005] was used to perform RT–qPCR on a StepOnePlus system [Applied Biosystems]. Each sample was duplicated (qPCR technical replica). The PCR cycle was 95 °C for 2 min followed by 40 cycles of 95 °C for 5 s and 60 °C for 30 s. For one experimental replicate, we averaged the cycle threshold (*Ct*) values of 4 biological replicates (groups of 10 larvae). The relative expression of the gene of interest (GOI) was calculated as 2^−ΔΔCt^, where ΔΔ*Ct* = (*Ct*_*GOI(Treatment)*_ − *Ct*_*RpL32(Treatment)*_) − (*Ct*_*GOI(Control)*_ − *Ct*_*RpL32(Control)*_).

### Statistical analysis

The effects of different treatments on oil droplet melanization were analyzed with a quasibinomial generalized linear model, with the ratio of melanized to nonmelanized oil droplets as a response and treatment as a fixed effect. We used Tukey’s honest significant difference test to compare treatments. To test differences in lamellocyte numbers with different treatments, we used one-way ANOVA with Tukey’s test to compare treatments. We compared gene expression (2^−ΔΔCt^) using a two-tailed *t* test, correcting *P* values with the Bonferroni method.

We used a phylogenetic mixed model to analyze the effect of extracts of 44 insect species on oil droplet melanization. This allowed us both to reconstruct ancestral states across a phylogeny and to test whether *Drosophila* has evolved to specifically recognized parasitoid wasps after correcting for the confounding effect of the insect phylogeny. The ratio of melanized to nonmelanized oil droplets was the binomial response variable. Whether the insect was a parasitoid was treated as a fixed effect. The phylogeny was treated as a random effect, which allows the correlation between two species to be inversely proportional to the time since those species shared a common ancestor (following a Brownian model of evolution). A residual variance allowed for differences between species that are unrelated to the phylogeny. We used the phylogeny of the 44 insect species available through TimeTree [35]. The model was fitted using a Bayesian approach using MCMCglmm [60] using an inverse gamma prior. We ran 10^6^ burn-in iterations followed by 10^7^ iterations, sampling every 10^4^ iterations.

R v3.6/4 [61] and RStudio v1.2.5042 [62] were widely used for generating figures.

### Supplementary Information

Additional file 1: Figure S1. Treatment of wasp homogenate with pronase and proteinase K. Figure (.PDF) showing the effect of protease digestion on wasp homogenate ability to induce the melanization response in *D. melanogaster* larvae.

Additional file 2: Figure S2 Effect of autoclaving wasp homogenate before proteinase K treatment. Figure (.pdf) showing that autoclaving of wasp homogenate makes it more susceptible to protenase K treatment.

Additional file 3: Table S1 Quality and read mapping metrics for the 12 hemocyte and 12 fat body samples. Table (.xlsx)

Additional file 4: Table S2 Gene Ontology (GO) terms, KEGG/Reactome pathways and Interpro protein that were significantly enriched in the fat body genes upregulated in wasp homogenate compared to unchallenged homogenate. Table (.csv)

Additional file 5: Figure S3 Volcano plots contrasting log_2_ fold change in gene expression against *P*-values generated from differential expression tests, for hemocyte and fat body samples. Figure (.pdf)

Additional file 6: Figure S4 Heatmap of lamellocyte marker genes. Table (.csv)

Additional file 7: Table S3 Gene Ontology (GO) terms, KEGG/Reactome pathways and Interpro protein domains that are significantly enriched in the differentially expressed genes in wasp homogenate compared to unchallenged hemocytes.

Additional file 8: Table S4 List of primers used for qPCR.

## Data Availability

All data generated or analyzed during this study are included in this published article, its supplementary information files, and publicly available repositories. Raw and processed data files used to generate figures and the lists of differentially expressed genes are available in the NERC EDS Environmental Information Data Centre (https://doi.org/10.5285/06ea87f3-476d-40fd-acce-e6923e786d48) (63). Scripts to analyze data are available on GitHub (DOI: https://doi.org/10.5281/zenodo.7692031). Paired-end reads from oil and wasp injections were deposited into the NCBI Sequence Read Archive (SRA) and can be accessed with Bioproject ID PRJNA685781.

## Abbreviations

PAMP: Pathogen-associated molecular pattern
DAMP: Damage-associated molecular pattern
PPO2 and PPO3: Pro-phenoloxidase 2 and 3
TEP: Thioester-containing protein
scRNA-seq: Single-cell RNA sequencing
SAMP: Self-associated molecular pattern
