# Supplementary figures and images for: Recognition of nonself is necessary to activate Drosophila’s immune response against an insect parasite
Source: BMC Biol. 2024 Apr 22;22:89. doi: 10.1186/s12915-024-01886-1 (PMC11034056; doi:10.1186/s12915-024-01886-1)

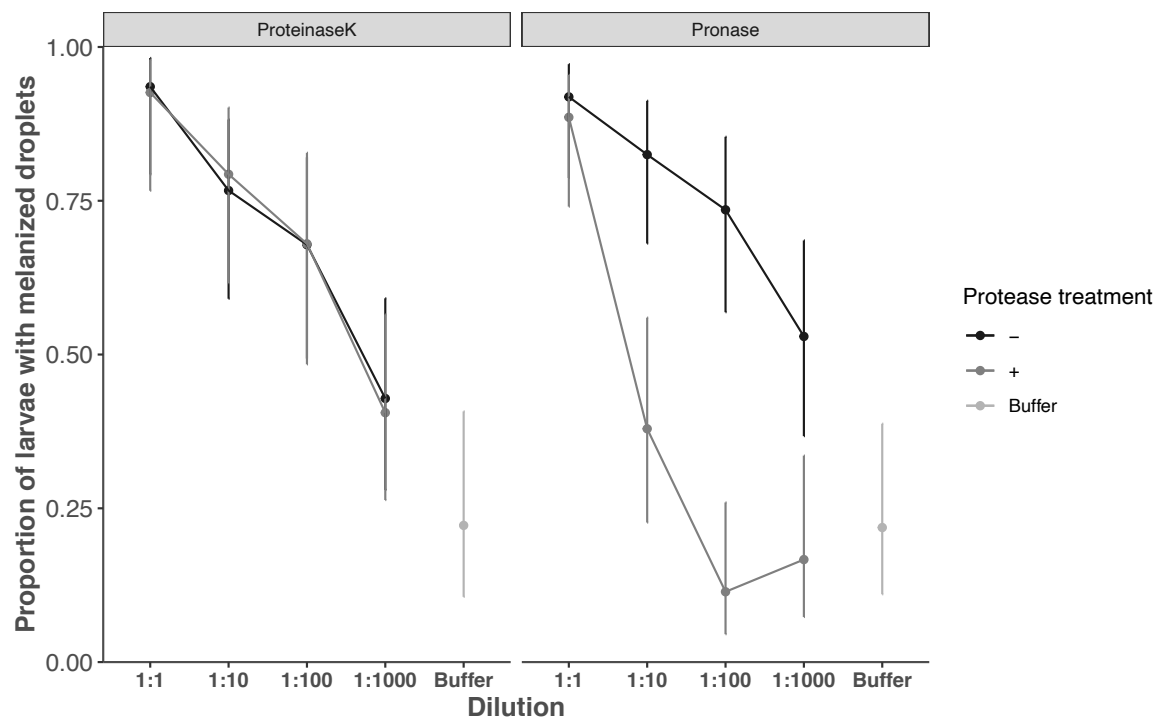

Supplement: Supplementary file 1 — Additional file 1: Figure S1. Treatment of wasp homogenate with pronase and proteinase K. Figure (.PDF) showing the effect of protease digestion on wasp homogenate ability to induce the melanization response in D. melanogaster larvae. [file 12915_2024_1886_MOESM1_ESM.pdf]

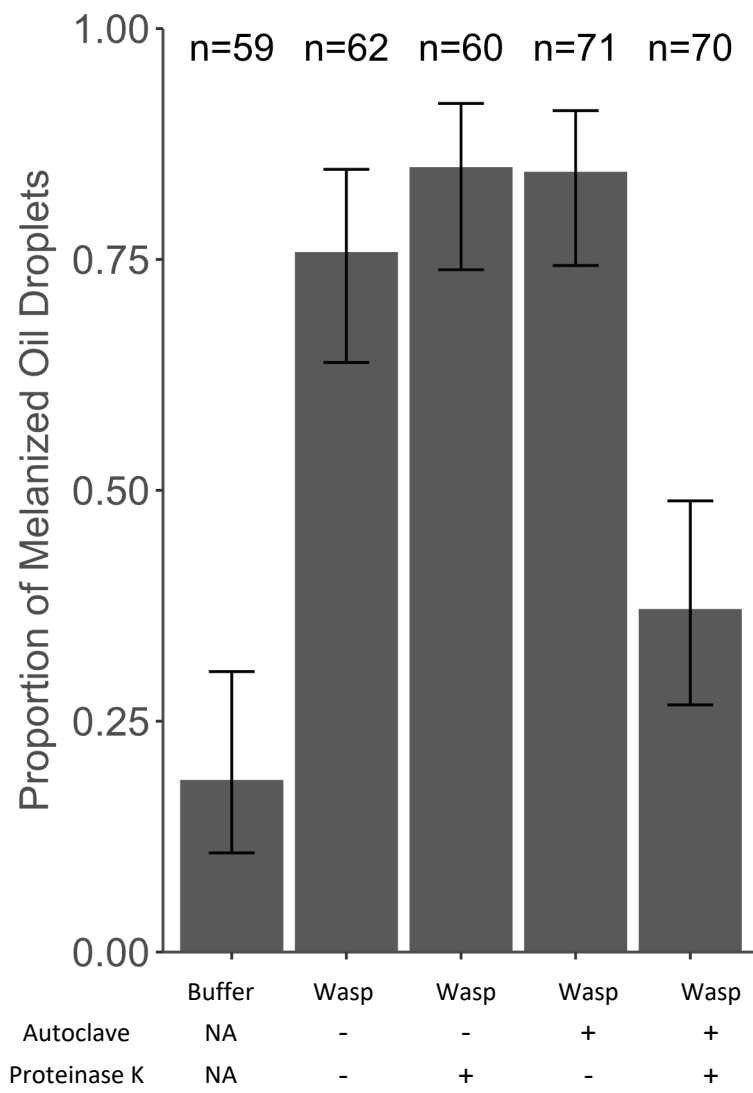

Supplement: Supplementary file 2 — Additional file 2: Figure S2 Effect of autoclaving wasp homogenate before proteinase K treatment. Figure (.pdf) showing that autoclaving of wasp homogenate makes it more susceptible to protenase K treatment. [file 12915_2024_1886_MOESM2_ESM.pdf]

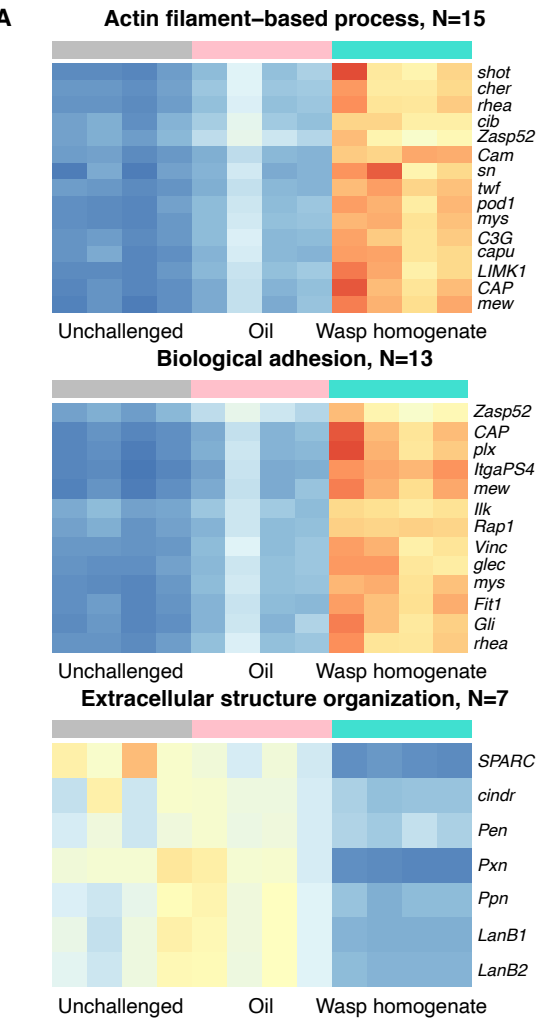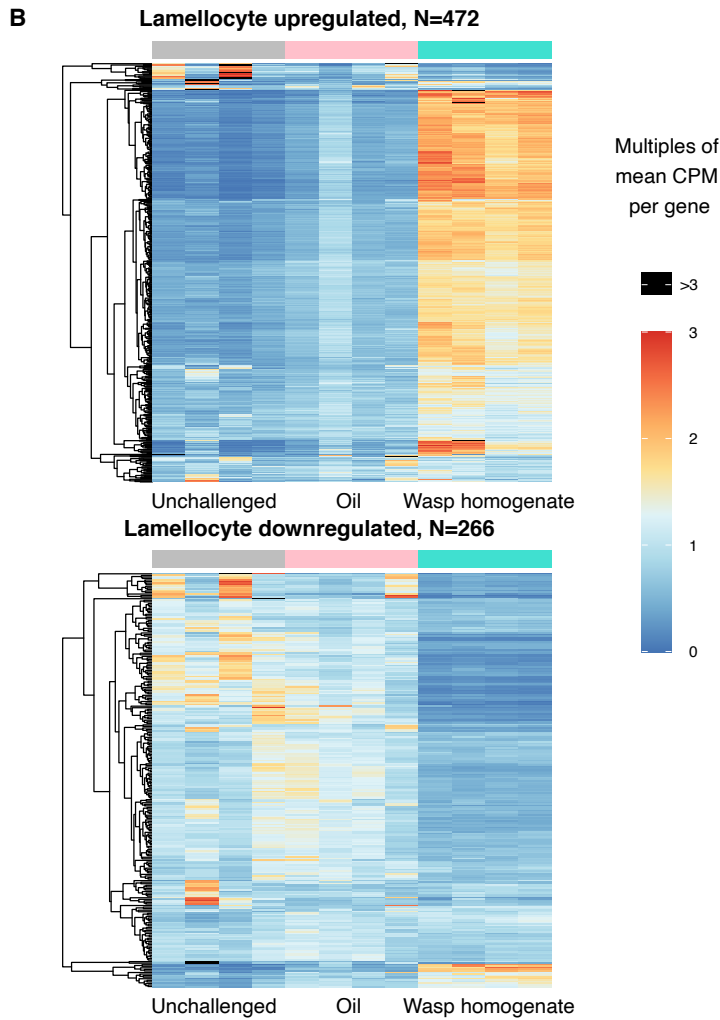

Supplement: Supplementary file 6 — Additional file 6: Figure S4 Heatmap of lamellocyte marker genes. Table (.csv) [file 12915_2024_1886_MOESM6_ESM.pdf]
