# Supplementary material for: Recognition of nonself is necessary to activate Drosophila’s immune response against an insect parasite
Source: BMC Biol. 2024 Apr 22;22:89. doi: 10.1186/s12915-024-01886-1 (PMC11034056; doi:10.1186/s12915-024-01886-1)
Supplement: Supplementary file 5 — Additional file 5: Figure S3 Volcano plots contrasting log2 fold change in gene expression against P-values generated from differential expression tests, for hemocyte and fat body samples. Figure (.pdf) [file 12915_2024_1886_MOESM5_ESM.pdf]

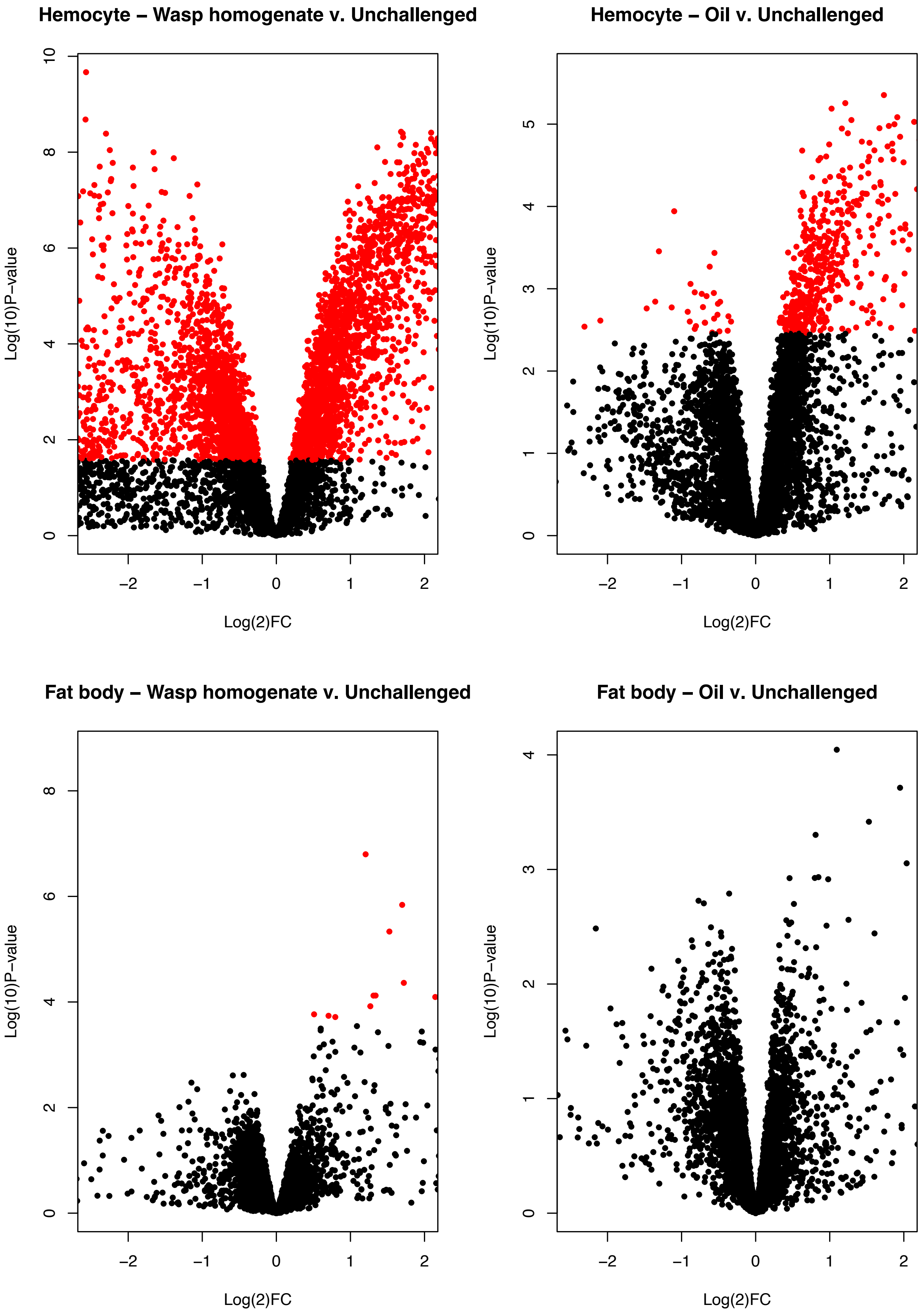

Hemocyte – Wasp homogenate v. Unchallenged
Hemocyte – Oil v. Unchallenged
Fat body – Wasp homogenate v. Unchallenged
Fat body – Oil v. Unchallenged
Log(10)P–value
Log(2)FC
0
2
4
6
8
10
–2
–1
1
3
5
